# The Weevil Genus *Rhamphus* (Curculionidae, Curculioninae) in Southern Africa—Description of Thirteen New Species

**DOI:** 10.3390/insects16050454

**Published:** 2025-04-25

**Authors:** Roberto Caldara, Michele Tedeschi

**Affiliations:** 1 Independent Researcher, Via Lorenteggio 37, IT-20146 Milano, Italy; 2 Independent Researcher, Via Paolo Giovio 19, IT-20144 Milano, Italy; michele.tedeschi2@gmail.com

**Keywords:** coleoptera, rhamphini, taxonomy, new species, bionomics, distribution

## Abstract

The southern African species of the genus *Rhamphus* Clairville, 1798, is herein revised for the first time. Fourteen species are recognized, 13 of which are new to science. The single known species was *Rhamphus namibicus* Korotyaev, 1994 (Namibia), whereas the new species are *R. carinatus* sp. nov. (South Africa: Limpopo; Zimbabwe), *R. densepunctatus* sp. nov. (South Africa: Western Cape), *R. gigas* sp. nov. (Zambia), *R. glaber* sp. nov. (South Africa: Mpumalanga), *R. globipennis* sp. nov. (South Africa: Mpumalanga, Limpopo, KwaZulu-Natal, Eastern Cape, Western Cape), *R. hispidulus* sp. nov. (South Africa: Eastern Cape), *R. indifferens* sp. nov. (South Africa: Gauteng, KwaZulu-Natal, Eastern Cape), *R. levipennis* sp. nov. (South Africa: Mpumalanga; Zimbabwe), *R. longitarsis* sp. nov. (Southern Namibia; South Africa: Northern Cape), *R. obesulus* sp. nov. (South Africa: Eastern Cape), *R. pilosulus* sp. nov. (South Africa: Mpumalanga, KwaZulu-Natal; Zimbabwe), *R. scaber* sp. nov. (South Africa: KwaZulu-Natal, Eastern Cape), *R. squamidorsum* sp. nov. (South Africa: Eastern Cape, Western Cape). Based on morphological characters, the species are separated into four informal groups. In considering possible relationships between these species and those from other regions, host-plant associations are also discussed.

## Simple Summary

Within the Rhamphini, the genus *Rhamphus* Clairville, 1798, comprises some of the smallest weevil species, including several taxa with a length of around 1 mm. This tribe is characterized by broad metafemora, modified for jumping, and belongs to the large subfamily Curculioninae of the family Curculionidae. The genus *Rhamphus* occurs in the Palaearctic, Afrotropical, Oriental, and Australian regions. This paper deals with the poorly known southern African species for the first time, focusing on morphological characters. Previously, a single species of *Rhamphus* was known from southern Africa. We now describe 13 new species, from Zimbabwe, Namibia, and South Africa. Illustrations of habitus and male genitalia are provided, and there are notes on biologies and a key to all of the included species.

## 1. Introduction

The leaf-miner weevil genus *Rhamphus* Clairville, 1798 belongs to the tribe Rhamphini (Curculionidae, Curculioninae), which is characterized by large metafemora, modified for jumping. Presently this genus comprises about thirty species: fifteen are Palaearctic, one is from the Oriental region (Sri Lanka), eight are from the Australian region, and finally, four are from the Afrotropical region, two of which are from Ethiopia, one from Sudan, and one from Namibia [1,2,3,4,5,6,7,8,9,10,11].

Recently we had the opportunity to study a large number of specimens of *Rhamphus* from southern Africa. A careful morphological study of this material allowed us to separate thirteen new species; the description of these is the purpose of the present paper.

## 2. Materials and Methods

### 2.1. Samples

We studied about 200 specimens of *Rhamphus* collected mainly in South Africa but also in Namibia, Zimbabwe, and Zambia and deposited in public institutions and private collections (see Depositories Section 2.4). Moreover, for comparison we examined many specimens of mostly Palaearctic species and several Australian specimens.

Labels are reported verbatim; additions, modifications in the name of geographical localities, explanations of the abbreviations, when necessary, and spelling corrections are in [square brackets]. All specimens have a further red label with the indication “HOLOTYPE (or PARATYPE) Rhamphus (species name) n. sp. det. Caldara & Tedeschi 2025”.

### 2.2. Measurements

All the specimens studied were measured. Measurements were made using an ocular micrometer with a Wild M8 stereoscopic microscope. Body length was measured from the anterior margin of the pronotum along the midline to the apex of the elytra (in this measurement, we excluded the length of the head because of variation due to its inclination). The length of the pronotum (Pl) was measured along the midline from the apex to the base, whereas its width (Pw) was measured across the widest point. The width of the pronotum was expressed as the ratio Pw/Pl. The length of the elytra (El) was measured along the midline from the transverse line joining the most anterior point of the humeri to the apex, whereas the width (Ew) was measured across the widest point. The proportions of the elytra were also expressed as the ratios El/Ew and Ew/Pw.

### 2.3. Description

In the diagnostic descriptions, we summarize the characters, which together enable the identification of each taxon, avoiding characters common to every species of the genus. No substantial differences between species were observed in the female genitalia (spermatheca and spiculum ventrale) of the species here considered, such as in the Palaearctic species [10], being similar to those already illustrated [9]. Due to the very small size of the specimens (the length of some species is less than 1 mm), the genitalia were only studied in species where more than five specimens were available to us; during dissection, the risk of serious damage to the specimen as a whole is high [5,6].

For terminology, we followed the online glossary of weevil characters proposed on the International Weevil Community Website (Available online: http://weevil.info/glossary-weevil-characters, accessed on 1 February 2025), edited by C.H.C. Lyal (British Museum of Natural History, London, UK).

### 2.4. Depositories

The names of the collections housing the material studied herein are abbreviated as follows:

BMNH British Museum of Natural History, London, UK (M. Barclay)

CBCP Centre de Biologie pour la Gestion des Populations, Montpellier, France (J. Haran)

ECPC Enzo Colonnelli, private collection, Roma, Italy

MCZR Museo civico di Zoologia, Roma, Italy (R. Casalini)

MKPC Michael Košťál, private collection, Šoporňa, Slovakia

MSNM Museo civico di Storia Naturale, Milan, Italy (F. Rigato, M. Zilioli)

MSNV Museo civico di Storia Naturale, Verona, Italy (L. Latella)

MTPC Michele Tedeschi, private collection Milan, Italy

RBPC Roman Borovec, private collection, Sloupno, Czech Republic

RCPC Roberto Caldara, private collection, Milan, Italy

### 2.5. Abbreviations in Descriptions

The abbreviations are as follows: E = elytra (elytral); l = length; P = pronotum (pronotal); w = width.

## 3. Results

### 3.1. Taxonomy

Curculionidae

Curculioninae

Rhamphini

***Rhamphus* Clairville**

*Rhamphus* [Clairville], 1798: pl. xii type species *Rhamphus flavicornis* [Clairville], 1798 nomen oblitum (=*Curculio oxyacanthae* Marsham, 1802 nomen protectum) [12]. Morimoto, 1984: 20 [13]. Kojima & Morimoto, 1996: 112, 114 [14]. Caldara et al., 2022: 375 [15]. Alonso-Zarazaga et al. 2023: 213 [11].

*Rhamphonyx* Voss, 1964: 592 type species *Rhamphonyx tarsalis* Voss, 1964 [3]. Colonnelli, 2009: 230 [6].

*Rhamphus* subgen. *Nanorhamphus* Korotyaev, 1984: 352 type species *Rhamphus emeljanovi* Korotyaev, 1984 [16]. Kojima & Morimoto, 1996: 114 [14]. Colonnelli, 2009: 230 [6].

*Rhamphus* subgen. *Trichorhamphus* Korotyaev, 1984: 351 type species *Rhamphus hisamatsui* Chûjô & Morimoto, 1960 [16]. Kojima & Morimoto, 1996: 114 [14]. Colonnelli, 2009: 230 [6].

*Diagnosis*. Head strongly bent between eyes. Rostrum usually retracted between forecoxae in dead specimens, as long as prosternum, in lateral view slightly curved and thin, in dorsal view with widest point at apex, with sparse lateral punctures; upper face at base distinctly convex, with two more or less distinct small tubercles close to each other at antennal insertion. Antennae inserted between forehead and base of rostrum, not geniculate, with oval scape as long as or slightly longer than first segment of funicle. Prosternum with coxae separated from one another. Mesosternal process and metasternum flat, the former about as wide as abdominal process between hind coxae. Venter with ventrites 2–4 straight in posterior margin. Femora unarmed, hind femora swollen. Fore- and midtibiae each armed with a hook-shaped uncus a little behind apex dorsally. Hind tibiae simple, unarmed at apex. Spermatheca with gland located at middle or beyond middle of capsule.

*Comparative notes*. Diagnostic characters were partly inferred from Kojima and Morimoto [14], who published a detailed phylogenetic study of the tribe Rhamphini based on morphological characters. This genus is easily distinguishable from the other genera of the tribe Rhamphini, mainly by the forehead being strongly angled between the eyes and prominent anteriorly. The rostrum is always retracted between forecoxae, the antennae inserted on the lateral surfaces of the prominence between eyes, not geniculate, and scrobes absent.

*Biological notes*. Species belonging to the genus *Rhamphus* are leaf miners in common with other Rhamphini. The mines are generally small, pear-shaped, and with half of their area stuffed with frass. Sometimes, more than one larva feed on the same leaf, producing several mines. Larvae hibernate in the fallen leaf and pupate in the same mine. Prior to oviposition, the adult feeding on the leaves causes a large number of tiny holes. Many *Rhamphus* host-plant species are well known and belong to particular families. In the Palaearctic region, the association is with Rosaceae (mainly *Prunus* and *Crataegus*), Salicaceae (*Salix*, *Populus*), Betulaceae (*Betula*), and Cistaceae (*Halimium*). In the Afrotropical, Oriental, and Australian regions, the association is with Leguminosae (=Fabaceae), mainly of the subfamily Caesalpinioideae. The species are usually monophagous and do not generally feed on plants belonging to different families.

*Distribution*. This genus is known to occur from the Palaearctic, Afrotropical, Oriental, and Australian Regions.

#### 3.1.1. List of Treated Species

A. *Rhamphus glaber* group
  1. *Rhamphus glaber* sp. nov.
  2. *Rhamphus longitarsis* sp. nov.

B. *Rhamphus pilosulus* group
  3. *Rhamphus pilosulus* sp. nov.
  4. *Rhamphus densepunctatus* sp. nov.
  5. *Rhamphus scaber* sp. nov.
  6. *Rhamphus carinatus* sp. nov.
  7. *Rhamphus gigas* sp. nov.
  8. *Rhamphus obesulus* sp. nov.
  9. *Rhamphus squamidorsum* sp. nov.

C. *Rhamphus levipennis* group
  10. *Rhamphus levipennis* sp. nov.
  11. *Rhamphus indifferens* sp. nov.
  12. *Rhamphus globipennis* sp. nov.

D. *Rhamphus hispidulus* group
  13. *Rhamphus hispidulus* sp. nov.

#### 3.1.2. Treatment of the Species

**A. ***Rhamphus glaber*** group** (two tarsal claws with appendices, integument without scales on dorsum).

**Figure 1:**
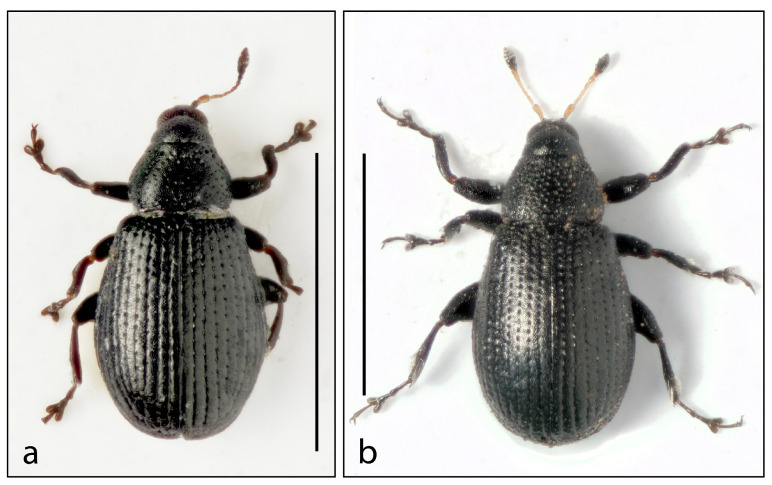
Habitus. (**a**) *Rhamphus glaber* **sp. nov.** and (**b**) *Rhamphus longitarsis* **sp. nov**. Scale bar: 1 mm.

1. ***Rhamphus glaber*** **sp. nov.** (Figure 1a and Figure 5a)

LSID urn:lsid:zoobank.org:act:75CB84CB-EFB5-4608-BE68-07DB6F0CA672

*Type locality*. Blydepoort (Mpumalanga, South Africa).

*Type series*. Holotype ♂: “SUD AFRICA, Transvaal [currently Mpumalanga]- Blydepoort, 20 - XI - 1981 Klapperich” (MSNV). Paratypes: same data as holotype (6, MTPC, RCPC).

*Diagnostic description*. Holotype. Length 0.9 mm. Integument shining black except for funicular segments of antennae being reddish (scape and club black). Rostrum in dorsal view with small protuberance at antennal insertion. Antennae short with clavate scape, 1.5× longer than wide and half the length of first funicular segment, which is as robust as scape, 3.0× longer than wide and distinctly more robust than others, clavate, 2.5× longer than second segment, which is 1.3× longer than wide, third–fifth segments 1.2× longer than wide, sixth–seventh segments transverse, latter very close to club. Eyes convex. Prothorax moderately transverse (Pw/Pl 1.50), widest in basal half, with moderately rounded sides; pronotum with rectilinear anterior margin, with moderately dense punctures, intervals between punctures as large as or larger than punctures and with opaque surface and distinct microsculpture. Elytra suboval, moderately longer than wide (El/Ew 1.25), with slightly pronounced humeri, at base slightly wider than pronotum, widest behind middle (Ew/Pw 1.48), weakly convex; striae with distinct punctures being separated from each other by narrow intervals placed on same plain as interstriae, the latter as wide as striae, flat and without scales. Metafemora distinctly globose (l/w 2.5). Pro- and mesotibiae with small uncus (l/w 2.5). First tarsomere 2.0× longer than wide, second tarsomere as long as wide, onychium 4.0× longer than wide, claws small, symmetrical, with indistinct appendices. Body of penis widest at base, then gradually narrowing and subparallel-sided in apical half, with blunted tip, more sclerified at middle with numerous small spine-like sclerites and with two flattened lateral orificial sclerites.

Variability. Length 0.8–1.0 mm. No remarkable differences between the specimens of the type series.

*Etymology*. The name of this species refers to the lack of vestiture of the dorsal integument.

*Comparative notes*. Among the species with two claws, *R. glaber* and *R. longitarsis* are unique, without pubescence. However, the first and second tarsomeres and the onychium in *R. glaber* are distinctly shorter than in *R. longitarsis* and similar to those of all the other species of this group. Moreover, *R. glaber* is smaller than *R. longitarsis* (0.8–1.0 mm vs. 1.2–1.3 mm).

*Biological notes*. No data are available.

*Distribution*. South Africa (Mpumalanga).

2. ***Rhamphus longitarsis*** **sp. nov.** (Figure 1b)

LSID urn:lsid:zoobank.org:act:AF31466B-F9D4-4C34-867D-47186B089514

*Type locality*. Aus (Namibia).

*Type series*. Holotype ♀: “Aus. 8-30.xi.1929/S.W. Africa. [currently Namibia] R.E.Turner. Brit. Mus. 1930–113” (BMNH). Paratypes: same data as holotype (1 ♀, RCPC); “SOUTH AFRICA, NORTHERN CAPE bor.or., Michael Košťál leg./Maheane, pr. Kuruman 1300 m, S 27°22.2′ E 23°17.3′, 22.x.2023” (1, MKPC).

*Diagnostic description*. Holotype. Length 1.2 mm. Integument shining black except for funicular segments of antennae being reddish (scape and club black). Rostrum in dorsal view with small protuberance at antennal insertion. Antennae with clavate scape, 1.5× longer than wide and half the length of first funicular segment, which is as robust as scape, 3.0× longer than wide and distinctly more robust than others, clavate, 2.5× longer than second segment, which is 1.5× longer than wide, third and fourth segments 1.3× longer than wide, fifth–seventh segments transverse. Eyes convex. Prothorax slightly transverse (Pw/Pl 1.36), widest in basal half, with moderately rounded sides; pronotum with rectilinear anterior margin, with dense punctures, intervals between punctures as large as or larger than punctures and with opaque surface and distinct microsculpture. Elytra suboval, distinctly longer than wide (El/Ew 1.33), with slightly pronounced humeri, at base slightly wider than pronotum, widest behind middle (Ew/Pw 1.50), weakly convex; striae with distinct punctures being separated from each other by narrow intervals placed on a same plain as interstriae, the latter as wide as striae, flat and without scales. Metafemora distinctly globose (l/w 2.5). Pro- and mesotibiae with small uncus (l/w 2.5). Protarsi and mesotarsi with first tarsomere 2.5× longer than wide, second tarsomere 1.5× longer than wide, onychium long, 8.0× longer than wide, metatarsi with first and second tarsomere longer respectively 6.0× and 3.0× longer than wide, claws small, symmetrical, with small appendices joined to claw.

Variability. Length 1.2–1.3 mm. No remarkable differences between the three specimens of the type series.

*Etymology*. The name of this species refers to its long tarsomeres, especially those of the hind legs.

*Comparative notes*. Among the species with two claws, *R. longitarsis* and *R. glaber* are unique, without pubescence. However, in *R. longitarsis* the first and second tarsomeres and the onychium of the metatarsi are distinctly longer than in *R. glaber*—which is also smaller (0.9–1.0 mm vs. 1.2–1.3 mm)—as well as in all the other species here considered.

*Biological notes*. No data are available.

*Distribution*. Southern Namibia, South Africa (Northern Cape).

**B. *Rhamphus pilosulus* group** (two tarsal claws with appendices, integument with more or less thin scales on dorsum)

**Figure 2:**
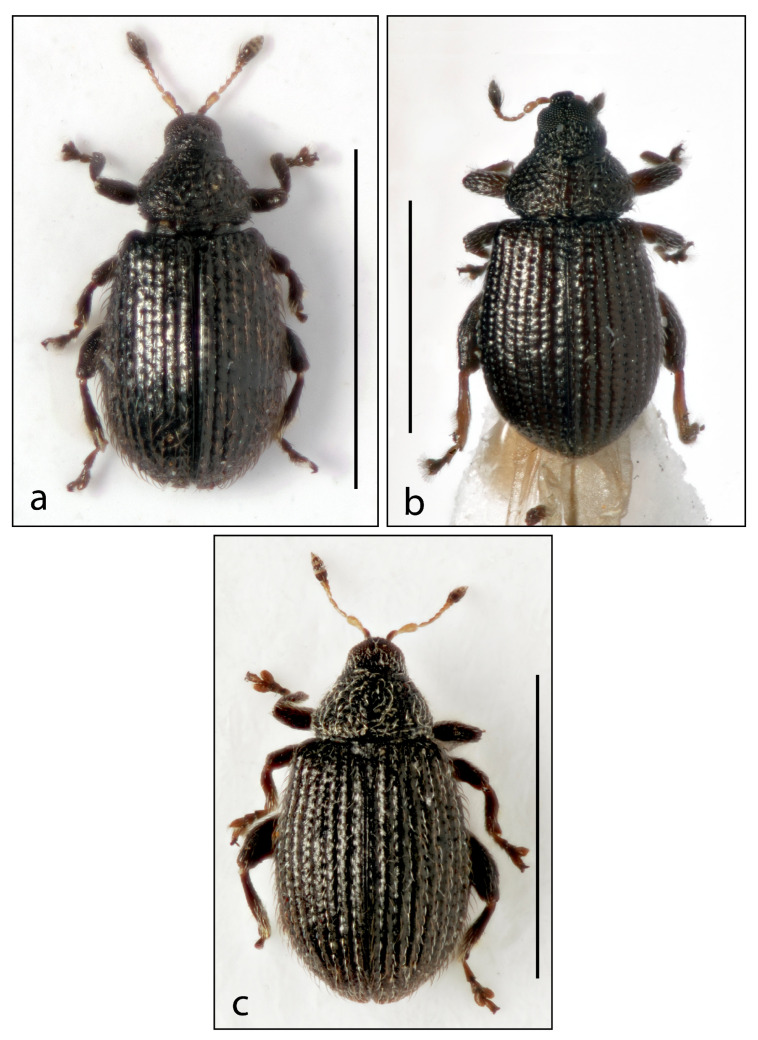
Habitus. (**a**) *Rhamphus pilosulus* **sp. nov.**, (**b**) *Rhamphus denspunctatus* **sp. nov.**, and (**c**) *Rhamphus scaber* **sp. nov.** Scale bar: 1 mm.

3. ***Rhamphus pilosulus***  **sp. nov.** (Figure 2a and Figure 5b)

LSID urn:lsid:zoobank.org:act:92724F1D-6698-4BA7-8D00-1F6740CCA20A

*Type locality*. Blydepoort (Mpumalanga, South Africa).

*Type series*. Holotype ♂: “SUD AFRICA, Transvaal [currently Mpumalanga] - Blydepoort, 20 - XI - 1981, Klapperich” (MSNV). Paratypes: same data as holotype (43, MSNM, MTPC, RCPC); “NATAL [currently KwaZulu-Natal], Weenen. IV.1929, H.P.Thomasset/Pres. By Imp. Bur. Ent. Brit. Mus. 1929–407” (1, BMNH); “Zimbabwe 1380 m, Shanghani farm, 19°41.746′ S, 29°19.117′ E, 7.xii.2017 R. Borovec lgt./Beating on shrubs and trees, mopane forest” (1, RBPC).

*Diagnostic description*. Holotype. Length 1.0 mm. Integument shining black except for funicular segments of antennae being reddish (scape and club darker than funicular segments). Rostrum in dorsal view with small protuberance at antennal insertion. Antennae with clavate scape, twice as long as wide and slightly shorter than first funicular segment, which is more robust than scape, 2.5× longer than wide and distinctly more robust than others, clavate, 1.6× longer than second segment, which is 1.3× longer than wide, third and fourth segments 1.2× longer than wide, fifth–seventh segments transverse. Eyes convex. Prothorax moderately transverse (Pw/Pl 1.40), widest at middle, with rounded sides; pronotum with rectilinear apical border, with large, deep, and dense punctures, with recumbent whitish hair-like (l/w 8–10) scales, intervals between punctures a little narrower than punctures and with moderately shining surface and distinct microsculpture. Elytra suboval, distinctly longer than wide (El/Ew 1.32), with pronounced humeri, at base wider than pronotum, widest just behind middle (Ew/Pw 1.29), weakly convex; striae with distinct punctures being separated from each other by narrow intervals placed on a plain slightly lower than that of interstriae, the latter as wide as striae, slightly convex and covered with recurved, subrecumbent, subtle (l/w 10–15) blackish hair-like scales arranged in a regular single row. Metafemora distinctly globose (l/w 2.5). Pro- and mesotibiae with small uncus (l/w 3). First tarsomere 3.5× longer than wide, second tarsomere 1.4× longer than wide, onychium short, 1.8× longer than wide, claws small, symmetrical, with two median appendices as long as half of claw, being closer to each other than to claw. Body of penis gradually enlarged from base to apex, with moderately blunted tip, more sclerotized at basal third, with a few sclerites at middle of internal sac and with two small flattened lateral orificial sclerites.

Variability. Length 0.9–1.1 mm. No relevant differences between the specimens of the type series.

*Etymology*. The name of this species refers to the vestiture of its elytral interstriae formed by hair-like scales.

*Comparative notes*. This taxon differs from the other species with two claws and vestiture with setae by the pronotum subrounded, slightly wider than long (vs. conical and distinctly transverse). Moreover, it can be separated from *R. scaber*, with which it shares a very small size, by the longer and more visible scales of the pronotum and longer elytra with slightly rounded sides and less convex interstriae.

*Biological notes*. The specimen from Zimbabwe was collected in a forest of *Colophospermum mopane* (J. Kirk ex Benth.) J. Léonard, a tree widely distributed in southern Africa and belonging to the family Leguminosae, subfamily Detarioideae, previously included in the Caesalpinioideae [17].

*Distribution*. Northeastern South Africa (Mpumalanga, KwaZulu-Natal), Zimbabwe.

4. ***Rhamphus densepunctatus***  **sp. nov.** (Figure 2b and Figure 5c)

LSID urn:lsid:zoobank.org:act:D912A5E5-CCA1-44D1-997C-1E2AD6A6637E 

*Type locality*. Mossel Bay (Western Cape, South Africa).

*Type series*. Holotype ♂: “S. Africa. R.E. Turner. Brit. Mus. 1921–210/Mossel Bay, Cape Province. April, 1921” (BMNH). Paratypes: same data as holotype (3, BMNH); “S. Africa. R.E. Turner. Brit. Mus. 1921–248/Mossel Bay, Cape Province. May, 1921” (2, BMNH) “S. Africa. R.E. Turner. Brit. Mus. 1921–294/Mossel Bay, Cape Province. Juin, 1921” (1, BMNH); “S. Africa. R.E. Turner. Brit. Mus. 1921–353/Mossel Bay, Cape Province. August, 1921” (1, BMNH) “S. Africa. R.E. Turner. Brit. Mus. 1921–412/Mossel Bay, Cape Province. Sept. 1921” (2, BMNH); “S. Africa. R.E. Turner. Brit. Mus. 1921–450/Mossel Bay, Cape Province. October, 1921” (15, BMNH, MTPC, RCPC); “S. Africa. R.E. Turner. Brit. Mus. 1930–402/Cape Province: Mossel Bay, Dec. 1934.” (1, BMNH); “S. Africa. R.E. Turner. B. M. 1930-73/Cape Province: Mossel Bay, vi-vii.1930” (1, BMNH); “ZA: W Cape - Tsitsikamma N.P., near Nature’s Valley, 33°57′95″ S 23°33′362″ E, 15.XI.2006, E. Colonnelli” (1, ECPC); “REP. OF SOUTH AFRICA, WC [Western Cape] Pr. Nature Valley, 26.vii.2019, J. Haran coll./JHAR03432 -33.981 23.564 battage, Collection-Cirad” (1, CBGP); “REP. OF SOUTH AFRICA, WC [Western Cape] Pr. Gansbaai, Grootbos, 13.x.2018, J. Haran coll./JHAR01590-01 -34.586 19.416 Battage fynbos, Collection-Cirad” (3, CBGP).

*Diagnostic description*. Holotype. Length 1.3 mm. Integument shining black except for funicular segments of antennae being reddish (scape and club darker than funicular segments). Rostrum in dorsal view with moderately distinct protuberance at antennal insertion. Antennae with clavate scape, 1.8× longer than wide and slightly longer than first funicular segment, which is globose, more robust than scape, 1.5× longer than wide and distinctly more robust than others, 1.6× longer than second segment, which is 1.5× longer than wide as well as third and fourth segments, fifth segment as long as wide, sixth and seventh segments transverse. Eyes convex. Prothorax transverse (Pw/Pl 1.50), widest in basal half, with rounded sides; pronotum with apical margin concave, with robust and dense punctures each with a suberect, subtle, short, indistinct, whitish hair-like scale, intervals between punctures a little narrower than punctures and with moderately shining surface and distinct microsculpture. Elytra suboval, moderately longer than wide (El/Ew 1.22), with moderately pronounced humeri, at base slightly wider than pronotum, widest just behind middle (Ew/Pw 1.43), weakly convex; striae with distinct punctures being separated from each other by narrow intervals placed on a plain slightly lower than that of interstriae, the latter as wide as striae, slightly convex and covered with recurved, recumbent, indistinct, subtle (l/w 7–10), whitish hair-like scales. Metafemora distinctly globose (l/w 2.5). Pro- and mesotibiae with robust short uncus. First tarsomere 3.0× longer than wide, second tarsomere as long as wide, onychium short, twice as long as wide, claws small, symmetrical, with indistinct appendices at the base. Body of penis with sides moderately rounded in basal third then parallel to apex, with blunted tip, without sclerites.

Variability. Length 1.2–1.4 mm. Many specimens of the type series collected in October 1921 have reddish elytra being immatures.

*Etymology*. The name of this species refers to the dense punctures of pronotum and elytral striae.

*Comparative notes*. This taxon is well separable from the other species with pubescent vestiture by the hair-like scales being shorter, recumbent, and poorly distinct on the elytra.

*Biological notes*. No data are available.

*Distribution*. South Africa (Western Cape).

5. ***Rhamphus scaber***  **sp. nov.** (Figure 2c)

LSID urn:lsid:zoobank.org:act:8201D507-6366-4A1B-9383-D782F85133A5

*Type locality*. St. Lucia (KwaZulu-Natal, South Africa).

*Type series*. Holotype ♀ “SUD AFRICA, NATAL [currently KwaZulu-Natal] S. LUCIA, 29-X-1981, Klapperich” (MSNV). Paratypes: same data of the holotype (2 ♀♀, MTPC, RCPC); “Natal: Kloof. 1500ft. Sept. 1926./S. Africa. R.E. Turner. Brit. Mus. 1926–404” (1, BMNH); “Port St. John[s]. Pondoland. Aug. 15–31. 1923./S. Africa. R.E. Turner. Brit. Mus. 1923–463” (2, BMNH).

*Diagnostic description*. Holotype. Length 1.1 mm. Integument shining black except for funicular segments of antennae being reddish (scape and club darker than funicular segments). Rostrum in dorsal view with distinct protuberance at antennal insertion.

Antennae with clavate scape, twice as long as wide and shorter than first funicular segment, which is slightly more robust than scape, twice as long as wide and distinctly more robust than others, clavate, 1.6× longer than second segment, which is 1.3× longer than wide, third and fourth segments 1.2× longer than wide, fifth–seventh segments transverse. Eyes convex. Prothorax subconical, distinctly transverse (Pw/Pl 1.70), widest at basal third, with moderately rounded sides; pronotum with concave anterior margin, with robust, deep, and dense punctures, irregularly arranged and with recumbent whitish hair-like (l/w 10–15) scales, intervals between punctures a little narrower than punctures and with subopaque surface and distinct microsculpture. Elytra subelliptical, moderately longer than wide (El/Ew 1.21), with rounded humeri, at base slightly wider than pronotum, widest just at middle (Ew/Pw 1.58), weakly convex; striae with distinct punctures being separated from each other by narrow intervals placed on a plain lower than that of interstriae, the latter as wide as striae, convex, rugulose, and covered with recurved, subrecumbent, subtle (l/w 10–15), whitish hair-like scales arranged in a regular single row. Metafemora distinctly globose (l/w 2.5). Pro- and mesotibiae with small uncus (l/w 3). First tarsomere 3.2× longer than wide, second tarsomere 1.2× longer than wide, onychium twice as long as wide, claws small, symmetrical, with two median appendices as long as half of claw and close to it.

Variability. Length 0.9–1.1 mm. The paratypes from the type locality do not differ from the holotype. The other paratypes have reddish elytra due to being immature.

*Etymology*. The name of this species refers to the rugulose sculpture of the pronotum and the elytral interstriae.

*Comparative notes*. This taxon is closely related to *R. pilosulus*, from which it differs by the conical and distinctly more transverse pronotum, which is covered with more distinct, longer, and broader scales, the longer elytra with more rounded sides and with more convex and more rugulose interstriae.

*Distribution*. Northeastern South Africa (KwaZulu-Natal, Eastern Cape).

**Figure 3:**
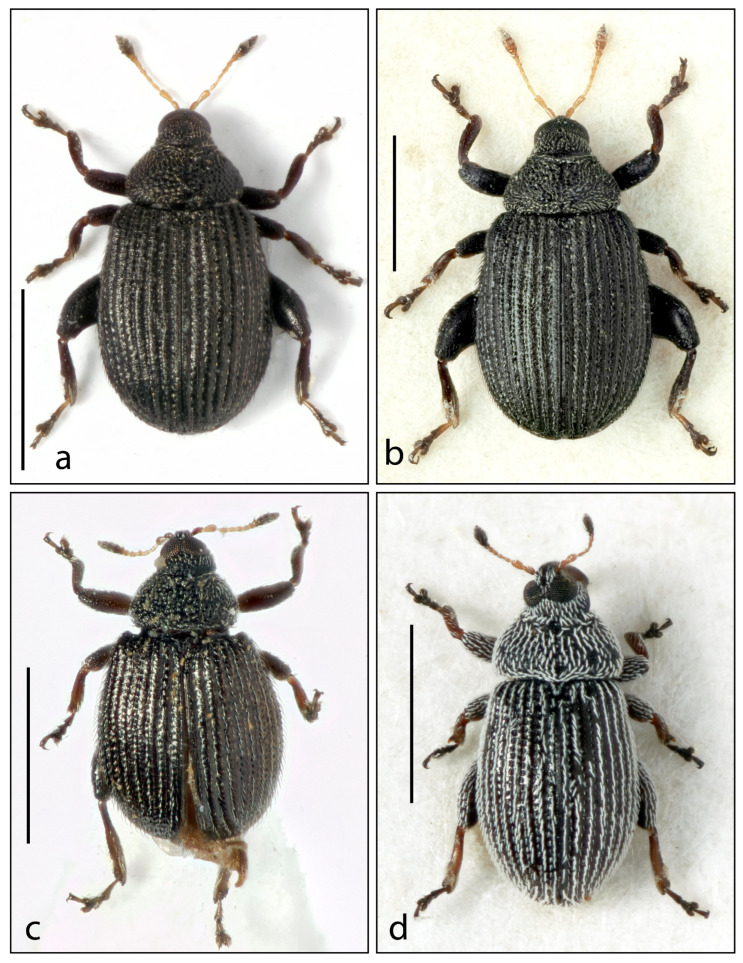
Habitus. (**a**) *Rhamphus carinatus* **sp. nov.**, (**b**) *Rhamphus gigas* **sp. nov.**, (**c**) *Rhamphus obesulus* **sp. nov.**, and (**d**) *Rhamphus squamidorsum* **sp. nov.** Scale bar: 1 mm.

6. ***Rhamphus carinatus***  **sp. nov.** (Figure 3a and Figure 5d)

LSIDurn:lsid:zoobank.org:act:46ABC6E1-4FA7-4984-A37F-A047E39D0B4B 

*Type locality*. Nylsvley Nature Reserve (Mookgophong, Limpopo, South Africa).

*Type series*. Holotype ♂: “NYLSVLEY, TVLE. [currently Limpopo] LEVEY 068/Burkea africana, 14.VII.76/S. Africa: Tvl. Naboomspruit [currently Mookgophong], Nylsvley PNR, 24°29′ S: 28°42′ E/B. Levey, B.M. 1980-29” (BMNH). Paratypes: as the holotype except “Burkea africana, 8.-9.1977” (1 ♀, BMNH); idem except “24/ii/1977” (1 ♀, BMNH); idem plus “Rhamphus sp. B. Levey 1976” (1 ♀, BMNH); “Zimbabwe 1313 m, Shanghani farm, 19°31.959′ S, 29°06.142′ E, 8.xii.2017 R.Borovec lgt./Beating on shrubs and trees, miombo forest” (2, RBPC, RCPC).

*Diagnostic description*. Holotype. Length 1.6 mm. Integument moderately shining black except for funicular segments of antennae being reddish yellow (club darker than funicular segments) and tibiae and tarsi being dark brown. Rostrum in dorsal view with distinct protuberance at antennal insertion. Antennae with clavate scape, 2.5× longer than wide and as long and as nearly robust as first funicular segment, which is 2.5× longer than wide and distinctly more robust than others, clavate, 1.3× longer than second segment, which is 1.8× longer than wide as well as third and fourth segments, fifth segment 1.2× longer than wide, sixth and seventh segments transverse. Eyes convex. Prothorax subconical, transverse (Pw/Pl 1.56), widest at basal half, with sides rounded and then rectilinear toward apex; pronotum with moderately concave anterior margin, with robust and dense punctures, each with a subrecumbent, subtle, short, indistinct, greyish hair-like scale, intervals between punctures a little narrower than punctures and with opaque surface and distinct microsculpture. Elytra subelliptical, moderately longer than wide (El/Ew 1.15), with slightly pronounced humeri, with slightly rounded sides, at base wider than pronotum, widest at middle (Ew/Pw 1.46), moderately convex; striae with poorly distinct punctures due to being separated from each other by narrow intervals placed on a plain distinctly lower than that of interstriae, the latter as wide as striae, distinctly convex and covered with recurved, subrecumbent, subtle (l/w 7–10), blackish hair-like scales. Metafemora distinctly globose (l/w 2.5). Pro- and mesotibiae with very short, robust uncus. First tarsomere of metatarsi long (4.5× longer than wide), that of protarsi 3.0× longer than wide, that of mesotarsi shorter (twice as long as wide), second tarsomere as long as wide, onychium short, 1.8× longer than wide, claws robust, symmetrical, with short appendices as long as one third of claw and closer to each other than to claw. Body of penis slightly and gradually narrowing from base to apex, with subacute tip, without sclerites.

Variability. Length 1.5–1.6 mm. The paratypes do not differ from the holotype.

*Etymology*. The name of this species refers to the distinctly convex elytral interstriae.

*Comparative notes*. It is somewhat similar to *R. gigas*, from which it differs by the smaller size and also by the more rounded sides of the elytra and the shorter antennae. Both species differ from the other species of the group by the opaque dorsal integument, the more flattened elytra, and the longer scape.

*Biological notes*. This species was collected on *Burkea africana* Hook. (Caesalpinioideae) in Limpopo province. It was also collected in “Miombo” forest, characterized by the dominant presence of *Brachystegia* and *Julbernardia*, genera of trees belonging to the family Leguminosae, subfamily Detarioideae, previously included in the Caesalpinioideae and distributed in the central and southern Africa [17].

*Distribution*. South Africa (Limpopo province), Zimbabwe.

7. ***Rhamphus gigas***  **sp. nov.** (Figure 3b and Figure 5e)

LSID urn:lsid:zoobank.org:act:16E10C7C-1C8C-4655-8496-DAFDE3749805

*Type locality*. Kashitu (Zambia).

*Type series*. Holotype ♂: “N.W. Rhodesia [currently Zambia]: Kashitu. N. of Broken Hill. VII.1915. H.C. Dollman./H.C. Dollman Coll. 1919–79” (BMNH). Paratypes: same data as the holotype (1 ♀, BMNH); idem except “2.VII.1915” (2 ♀♀, BMNH, RCPC).

*Diagnostic description*. Holotype. Length 2.0 mm. Integument moderately shining black except for funicular segments of antennae being reddish yellow (club darker than funicular segments) and tibiae and tarsi being dark brown. Rostrum in dorsal view with distinct protuberance at antennal insertion. Antennae with clavate scape, 3.0× longer than wide and as long and as nearly robust as first funicular segment, which is 3.0× longer than wide and distinctly more robust than others, clavate, 1.5× longer than second segment, which is 2.0× longer than wide as well as third and fourth segments, fifth and sixth segments 1.2× longer than wide, seventh segment as long as wide. Eyes convex. Prothorax subconical, transverse (Pw/Pl 1.67), widest at basal half, with sides rounded and then rectilinear; pronotum with distinctly concave anterior margin, with robust and dense punctures, each with a subrecumbent, subtle, short, indistinct, greyish hair-like scale, intervals between punctures a little narrower than punctures and with opaque surface and distinct microsculpture. Elytra subelliptical, moderately longer than wide (El/Ew 1.18), with slightly pronounced humeri, at base wider than pronotum, with distinctly rounded sides, widest at middle (Ew/Pw 1.58), moderately convex; striae with poorly distinct punctures due to being separated from each other by narrow intervals placed on a plain distinctly lower than that of interstriae, the latter as wide as striae, distinctly convex, and covered with recurved, subrecumbent, subtle (l/w 6–8), blackish hair-like scales. Metafemora distinctly globose (l/w 2.5). Pro- and mesotibiae with very short, robust uncus. First tarsomere of metatarsi long (4.5× longer than wide), that of protarsi 3.0× longer than wide, that of mesotarsi shorter (twice as long as wide), second tarsomere as long as wide, onychium short, 1.8× longer than wide, claws robust, symmetrical, with short appendices as long as one third of claw and closer to each other than to claw. Body of penis with parallel sides from base to near apex, with blunted tip, without sclerites.

Variability. Length 2.0–2.2 mm. The paratypes do not differ from the holotype.

*Etymology*. The name of this species refers to its size, which is bigger than all the other African species.

*Comparative notes*. Among all the taxa herein described, this is the biggest in length. It appears closely related to *R. carinatus*, from which it differs, apart from the size, by the more rounded sides of the elytra and the longer antennae.

*Biological notes*. No data are available.

*Distribution*. Known only from the type locality in Zambia.

8. ***Rhamphus obesulus***  **sp. nov.** (Figure 3c)

LSID urn:lsid:zoobank.org:act:8A7CCD98-04FE-4167-BBFB-9BFDBE7C7611

*Type locality*. Silaka Nature Reserve (Port St. Johns, Eastern Cape, South Africa).

*Type series*. Holotype ♀: “ZA: E Cape - Port St. Johns, Silaka Nature Reserve, 31.39.45 S 29.30.14 E, 10.XI.2006, E. Colonnelli” (MCZR).

*Diagnostic description*. Holotype. Length 1.5 mm. Integument shining black except for funicular segments of antennae being reddish (scape and club darker than funicular segments). Rostrum in dorsal view with distinct protuberance at antennal insertion. Antennae with clavate scape, 1.8× longer than wide and as long and as robust as first funicular segment, both distinctly more robust than others, clavate, 1.4× longer than second segment, which is 1.5× longer than wide, third segment 1.2× longer than wide, fourth–fifth segments as long as wide, other segments transverse. Eyes convex. Prothorax subconical, transverse (Pw/Pl 1.48), widest just before middle, with rounded sides; pronotum with sightly concave anterior margin, with robust and dense punctures, intervals between punctures narrower than punctures and with moderately shining surface and distinctly convex, with dense whitish recumbent seta-like scales. Elytra rounded, slightly longer than wide (El/Ew 1.12), with slightly pronounced humeri, at base wider than pronotum, widest at middle (Ew/Pw 1.62), moderately convex; striae with distinct punctures being separated from each other by narrow intervals placed on a plain lower than that of interstriae, the latter as wide as striae, distinctly convex, and covered with dense, recurved, subrecumbent, subtle (l/w 10–12), blackish hair-like scales. Metafemora distinctly globose (l/w 2.5). Pro- and mesotibiae with small uncus (l/w 3). First tarsomere 3.5× longer than wide, second tarsomere 1.4× longer than wide, onychium short, 2.0× longer than wide, claws small, symmetrical, with two median appendices, being half as long as the claw and close to it.

*Etymology*. The name of this species refers to the broad and globose shape of the elytra.

*Comparative notes*. This taxon differs from *R. carinatus* and *R. gigas* by the shining integument (vs. opaque), the more convex elytra (vs. flattened), and the dark brown scape (vs. reddish), and from *R. densepunctatus* by the more rounded elytra, the more distinct elytral scales, and the dark brown scape.

*Biological notes*. No data are available.

*Distribution*. South Africa (Eastern Cape).

9. ***Rhamphus squamidorsum***  **sp. nov.** (Figure 3d and Figure 5f)

LSID urn:lsid:zoobank.org:act:E93DDE30-EF8D-4C7E-AE6F-828BE89E7765

*Type locality*. Grahamstown (Eastern Cape, South Africa).

*Type series*. Holotype ♂: “ZA: E Cape – m 750, Grahamstown - Toposcope, 33.19.03 S 26.33.24 E, 13.XI.2006 - E. Colonnelli” (MCZR). Paratypes: same data of the holotype (5, ECPC, MTPC, RCPC); “Cape Province. Swellendam. 17.xii.31-18.i.32./S. Africa. R.E. Turner. Brit. Mus. 1932–56” (1, BMNH); “REP. OF SOUTH AFRICA, WC [Western Cape] Pr. Cholcester, 30.iv.2018, J. Haran coll./JHAR00950 -33.681 25.827 battage, Collection-Cirad” (5, CBGP); “Cape Province. Montagu. 23-30 Sept. 1924./S. Africa. R.E. Turner. Brit. Mus. 1924–547” (1, BMNH); “SOUTH AFRICA: W Cape, - 5 km S of Montagu - m 230, 33.48.12 S 20.06.02 E, 21.XI.2007 - E. Colonnelli” (2, ECPC); “RSA Western Cape 1002 m, Cedersberg Mts. Pakhuis Pass, 32°08.979′ S 19°01.750′ E, R.Borovec lgt. 22.xi.2016” (1, RBPC).

*Diagnostic description*. Holotype. Length 1.4 mm. Integument moderately shining, black except for antennae being reddish (club is dark brown as well tibiae and tarsi), dorsum and femora covered with moderately dense, recumbent, moderately elongate (l/w 4–6), suboval to subelliptical whitish scales, arranged in a single regular row on each interstria. Rostrum in dorsal view with small protuberance at antennal insertion. Antennae with globose scape, almost as long as wide and slightly shorter than first funicular segment, which is slightly more robust than scape, 1.5× longer than wide and distinctly more robust than others, globose, 1.4× longer than second segment, which is 1.3× longer than wide, third and fourth segments 1.2× longer than wide, fifth–seventh segments transverse. Eyes convex. Prothorax strongly transverse (Pw/Pl 1.87), widest at middle, with rounded sides; pronotum distinctly concave at apex, with robust and dense punctures well visible between scales, intervals between punctures a little narrower than punctures and with moderately shining surface and distinct microsculpture. Elytra suboval, distinctly longer than wide (El/Ew 1.25), with weakly pronounced humeri, at base as wide as pronotum, widest just behind middle (Ew/Pw 1.29), moderately convex; striae with distinct deep and broad punctures being separated from each other by narrow intervals placed on a plain slightly lower than that of interstriae, the latter narrower than striae, slightly convex. Metafemora distinctly globose (l/w 2.5). Pro- and mesotibiae with big robust uncus. First tarsomere 3.5× longer than wide, second tarsomere 1.4× longer than wide, onychium 2.5× longer than wide, claws robust, symmetrical with short small appendices completely joined to the claw. Body of penis with distinctly sinuous sides, being narrower at middle, with subacute tip, without sclerites.

Variability. Length 1.2–1.5 mm. The scales of the dorsum vary a little in their width. No other substantial differences between the specimens of the type series.

*Etymology*. The name, which means “with squamose dorsal surface”, highlights one of the most distinctive characters of this species.

*Comparative notes*. This taxon is easily distinguishable from all other species by the broader scales of the vestiture, which are not hair-like but rectangular to lanceolate. It is also noteworthy that the pronotum is strongly transverse and distinctly concave at its anterior margin.

*Biological notes*. No data are available.

*Distribution*. South Africa (Eastern Cape and Western Cape).

**C. *Rhamphus levipennis* group** (single tarsal claw, integument without scales on dorsum).

**Figure 4:**
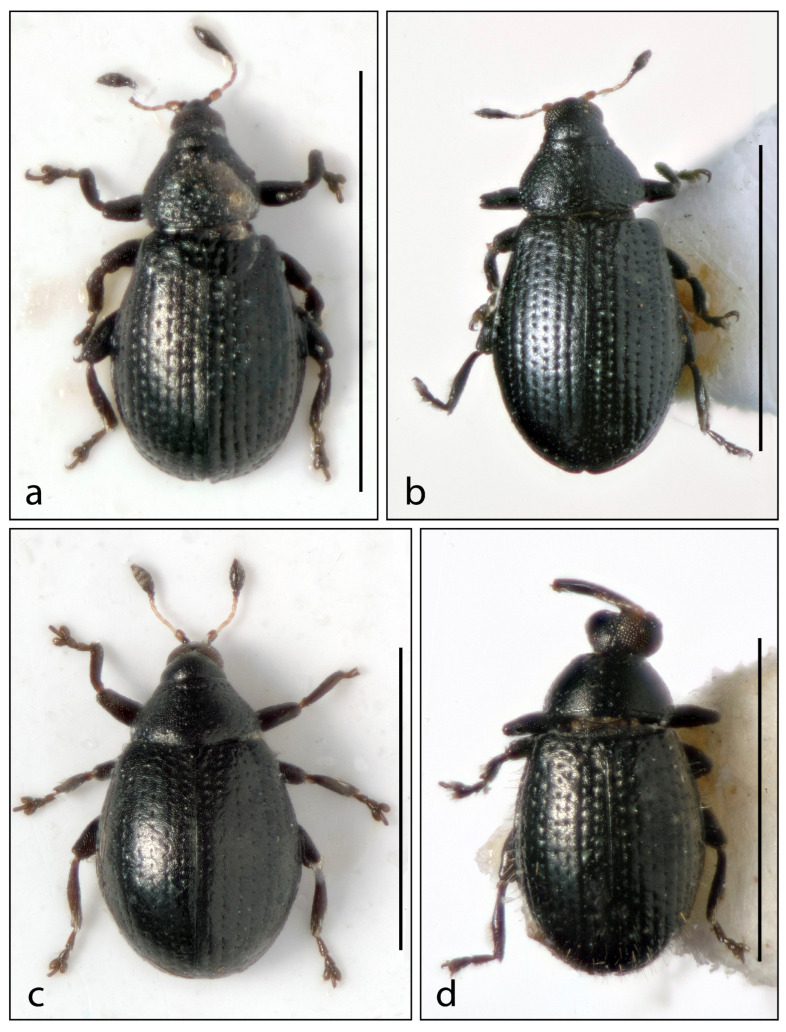
Habitus. (**a**) *Rhamphus levipennis* **sp. nov.**, (**b**) *Rhamphus indifferens* **sp. nov.**, (**c**) *Rhamphus globipennis* **sp. nov.**, and (**d**) *Rhamphus hispidulus* **sp. nov.** Scale bar: 1 mm.

**Figure 5:**
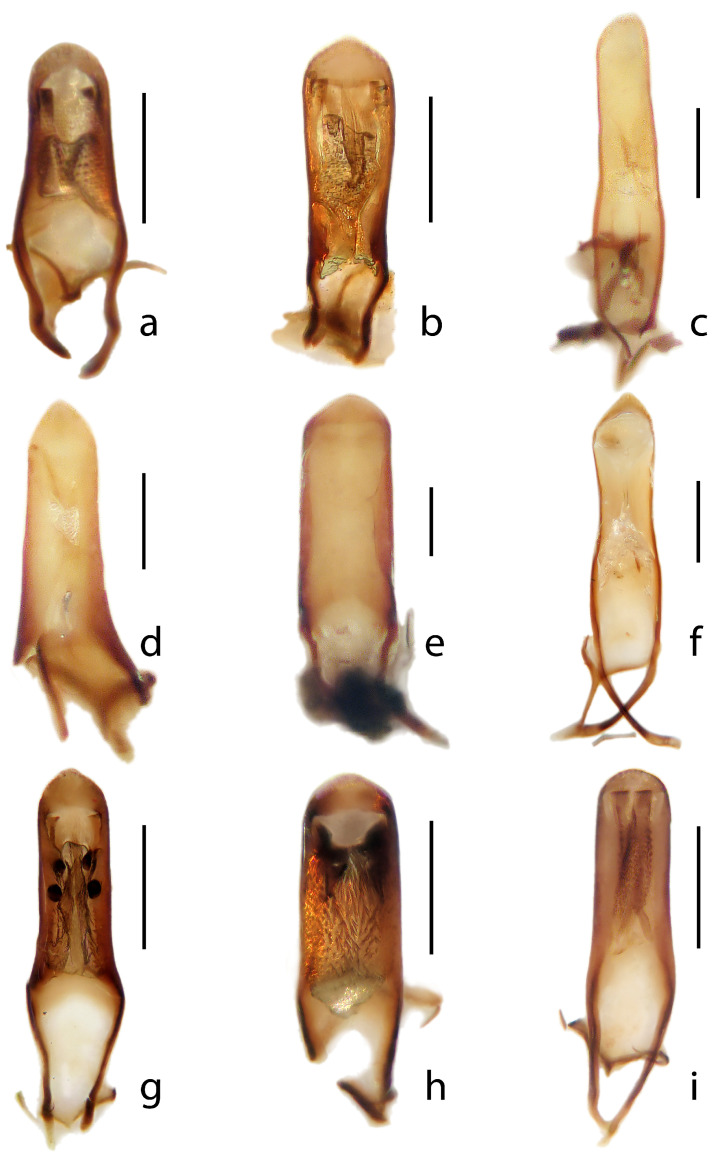
Penis in dorsal view. (**a**) *Rhamphus glaber* **sp. nov.**, (**b**) *Rhamphus pilosulus* **sp. nov.**, (**c**) *Rhamphus denspunctatus* **sp. nov.**, (**d**) *Rhamphus carinatus* **sp. nov.**, (**e**) *Rhamphus gigas* **sp. nov.**, (**f**) *Rhamphus squamidorsum* **sp. nov.** (**g**) *Rhamphus levipennis* **sp. nov.**, (**h**) *Rhamphus indifferens* **sp. nov.**, and (**i**) *Rhamphus globipennis* **sp. nov.** Scale bar: 0.1 mm.

10. ***Rhamphus levipennis***  **sp. nov.** (Figure 4a and Figure 5g)

LSID urn:lsid:zoobank.org:act:96F72E6F-DF7E-47D7-8A93-0A828F35BCC5

*Type locality*. Blydepoort (Mpumalanga, South Africa).

*Type series*. Holotype ♂: “SUD AFRICA, Transvaal [currently Mpumalanga]- Blydepoort, 20 - XI - 1981 Klapperich” (MSNV). Paratypes: same data as holotype (25, MSNM, MTPC, RCPC); “Sud Africa – Letaba, Kruger National Park, Transvaal [currently Mpumalanga] – 18.XI.1981, Leg. Klapperich” (1, MTPC); “REP. OF SOUTH AFRICA, KZN [KwaZulu-Natal] Pr., Hluhluwe 2.i.2019 J. Haran coll./JHAR02038-01 -28.025 32.348, sur *Acacia* fleurs jaunes Collection-Cirad” (2, CBGP); “Zimbabwe 1390 m, Shanghani farm, 19°41.746′ S, 29°19.117′ E, 7.xii.2017 R. Borovec lgt.” (1, RBPC).

*Diagnostic description*. Holotype. Length 0.8 mm. Integument shining black except for funicular segments of antennae being reddish (scape and club darker than funicular segments) and tarsi being brown. Rostrum in dorsal view with moderately distinct protuberance at antennal insertion. Antennae with globose scape, as long as wide and distinctly shorter than first funicular segment, which is more robust than scape, 1.6× longer than wide and distinctly more robust than others, cylindrical, as long as second segment, which is 1.7× longer than wide, third and fourth segments as long as wide, fifth–seventh segments transverse. Eyes convex. Prothorax weakly transverse (Pw/Pl 1.13), subrounded, widest at middle; pronotum with anterior margin rectilinear, with sparse, slightly deep punctures (denser and deeper in apical half), intervals between punctures distinctly broader than punctures and with moderately shining surface and distinct microsculpture. Elytra suboval, longer than wide (El/Ew 1.33), with weakly pronounced humeri, at base slightly wider than pronotum, widest just behind middle (Ew/Pw 1.48), weakly convex; striae with distinct punctures being separated from each other by large intervals placed on the same plain of interstriae, the latter as wide as striae, completely flat and without scales. Metafemora distinctly globose (l/w 2.5). Pro- and mesotibiae with small thin uncus (l/w 3). First tarsomere 3.0× longer than wide, second tarsomere 1.5× longer than wide, onychium thin, 2.5× longer than wide, claws robust, single. Body of penis wide at base, then narrowing and subparallel to near apex, with blunted tip, with distinct couples of sclerites at middle of internal sac and with two long flattened lateral sclerites ending at orifice.

Variability. Length 0.7–0.9 mm. No remarkable differences between the specimens of the type series.

*Etymology*. The name of this species refers to its flat elytral interstriae.

*Comparative notes*. It seems related to *R. indifferens*, from which it differs by the smaller size (length 0.7–0.9 vs. 1.0–1.2), the pronotum subrounded and not conical, and the punctures of the elytral striae sparser and with wider intervals between the punctures.

*Biological notes*. Some specimens of the type series were collected on *Acacia* sp. with yellow flowers (det. J. Haran).

*Distribution*. South Africa (Mpumalanga, KwaZulu-Natal), Zimbabwe.

11. ***Rhamphus indifferens***  **sp. nov.** (Figure 4b and Figure 5h)

LSID urn:lsid:zoobank.org:act:C13BCE43-0419-4CE8-9430-318B1260046E 

*Type locality*. Port St. Johns (Eastern Cape, South Africa).

*Type series*. Holotype ♂: “Port St. John[s]. Pondoland. July 1-9. 1923./S. Africa. R.E. Turner. Brit. Mus. 1923–369” (BMNH). Paratypes: “Port St. John[s]. Pondoland. April 1-5 1923./S. Africa. R.E. Turner. Brit. Mus. 1923–241” (1, BMNH); “Port St. John[s]. Pondoland. April 5-30. 1923./S. Africa. R.E. Turner. Brit. Mus. 1923–286” (1, BMNH); “Port St. John[s]. Pondoland. Aug. 15-31. 1923./S. Africa. R.E. Turner. Brit. Mus. 1923–463” (1, BMNH); Port St. John[s]. Pondoland. 1-17 Mar. 1924./S. Africa. R.E. Turner. Brit. Mus. 1924–177” (1, BMNH); “SOUTH AFRICA: E Cape, - 5 Km E Port St. Johns, 31.36.58S 29.34.61E, 8/9.XI.2006 - E. Colonnelli/Acacia sp. on leaves [green card]” (2, ECPC); “ZA: E Cape - road R61, - 10 Km NW Port St. Johns, 31°36′29″ S 29°27′95″ E, 7/9.XI.2006 - E. Colonnelli/Acacia, sp., on flowers [green card]” (2, ECPC, RCPC); “ZA: E Cape - m 280, road N2 - 35 Km W Peddie, 33°16′69″ S 26°48′95″ E, 7.XI.2006 - E. Colonnelli” (1, ECPC); “ZA: E Cape - Zuurberg, Pass road - m 550, 332166S 254456E, 17.XI. 2006 - E. Colonnelli/Acacia, sp. [green card]” (3, ECPC, MTPC); “Cape Province: Somerset East. Sept. 1930/S. Africa. R.E. Turner. Brit. Mus. 1930–480” (3, BMNH, RCPC); “Queenstown, Cape Province. 3500 ft. 16.i. – 10.ii. 1923./S. Africa. R.E. Turner. Brit. Mus. 1923–140” (1, BMNH); “Zululand [currently KwaZulu-Natal]: Empangeni. 26.iv.1926/S. Africa. R.E. Turner. Brit. Mus. 1926–194” (2, BMNH); “Zululand: Melmoth. 24.iv.1926./S. Africa. R.E. Turner. Brit. Mus. 1926–194” (1, BMNH). “REP. of South Africa, KZN [KwaZulu-Natal] Pr., Roodeplaat, 4.iii.2019, J. Haran coll./JHAR02259-01, -25.608 28.353, Battage *Vachellia karroo*, Collection – Cirad” (3, CBGP).

*Diagnostic description*. Holotype. Length 1.2 mm. Integument shining black except for funicular segments of antennae being reddish (scape blackish, first segment of funicle and club darker than other funicular segments). Rostrum in dorsal view with moderately distinct protuberance at antennal insertion. Antennae with globose scape, as long as wide and 1.3× shorter than first funicular segment, which is as robust as scape, 1.5× longer than wide and distinctly more robust than others, cylindrical, as long as second segment, which is 2.2× longer than wide, third–fifth segments 1.2× longer than wide, sixth and seventh segments transverse. Eyes convex. Prothorax transverse (Pw/Pl 1.48), widest just before middle, with rounded sides; pronotum with slightly concave anterior margin, with sparse and shallow punctures, intervals between punctures distinctly wider than punctures and with slightly shining surface and distinct microsculpture. Elytra subelliptical, moderately longer than wide (El/Ew 1.24), with weakly pronounced humeri, at base slightly wider than pronotum, widest just behind middle (Ew/Pw 1.45), weakly convex; striae with distinct punctures being separated from each other by narrow intervals placed on a same plain of that of interstriae, the latter as wide as striae, flat, without seta-like scales. Metafemora distinctly globose (l/w 2.5). Pro- and mesotibiae with small uncus (l/w 3). First tarsomere 3.5× longer than wide, second tarsomere 1.2× longer than wide, onychium short, 2.2× longer than wide, with single claw. Body of penis with parallel sides from base to near apex, with blunted tip, with symmetrical broad sclerites in apical third ending at orifice.

Variability. Length 1.0–1.2 mm. The width of the elytra is a little variable also in specimens of the same series (El/Ew 1.19–1.28).

*Etymology*. The name of this species is in its meaning “irrelevant” and refers to the lack of particular characters in this taxon.

*Comparative notes*. Among the species with a single row, it seems more closely related to *R. levipennis,* from which it differs by the slightly bigger size (length 1.0–1.2 vs. 0.7–0.9), the pronotum subconical and distinctly transverse, and the denser punctures of the elytral striae, with their diameter longer than intervals between the punctures.

*Biological notes*. Some specimens of the type series were collected on *Vachellia karroo* (det. J. Haran). Probably, also E. Colonnelli collected other specimens on the same plant, previously included in the genus *Acacia*.

*Distribution*. South Africa (Eastern Cape, KwaZulu-Natal, Gauteng).

12. ***Rhamphus globipennis***  **sp. nov.** (Figure 4c and Figure 5i)

LSID urn:lsid:zoobank.org:act:1D60A29F-E7D7-4E88-A8DD-504378F04E67

*Type locality*. Blydepoort (Mpumalanga, South Africa).

*Type series*. Holotype ♂: “SUD AFRICA, Transvaal [currently Mpumalanga] - Blydepoort, 20 - XI - 1981 Klapperich” (MSNV). Paratypes: same data as holotype (6, MSNM, MTPC, RCPC); REP. OF SOUTH AFRICA, MP [Mpumalanga] Pr., Nelspruit 06.iv.2018 J. Haran coll./JHAR00874-01 -25.445 30.970 battage Collection-Cirad” (3, CBGP); “REP. OF SOUTH AFRICA, LP Pr. Thohoyandou, 4.vii.2018, J. Haran coll./JHAR01137 -23041 30.462 Battage *Vachelia* sp., Collection-Cirad” (6, CBGP); “SUD AFRICA, NATAL [currently KwaZulu-Natal] S. LUCIA, 29 - X - 1981, Klapperich” (3, MTPC, RCPC); “ZA: E Cape - m 1000, 10 Km E of Whittlesea, 32.04.43S 26.47.35E, 12.XI.2006 - E. Colonnelli/Acacia, sp., on leaves [green card]” (10, ECPC, RCPC); “ RSA Eastern Cape 850 m 1 km W of Willowmore 33°17.4′ S 23°28.6′ E 7.xi.2023 R. Borovec lgt” (2, RBPC); “Cape Province: Somerset East. 1-9.xii. 1930/S. Africa. R.E. Turner. Brit. Mus. 1931–12” (1, BMNH); “Dumbrody E. Cape Prov. 9.1901 J. O’Neil/Pres. by Comm Inst Ent B.M. 1981–315/Rhamphus sp. nov.” (2. BMNH); “SOUTH AFRICA: W CAPE, - 25 Km N of Malgas - m 50, 34.11.43 S 20.34.16 E, 20.XI.2007 - E. Colonnelli/Acacia karoo Hayne [green card]” (5, ECPC); “Southern Cape: Western Cape: 25 km N Malgas m 150 20.XI.2007, Giusto, Colonnelli & Osella” (1, RCPC); “Sud Africa-Cape Province, De Rust valley - Karoo, 29.XI.1981, leg. Klapperich! (1, MTPC).

*Diagnostic description*. Holotype. Length 1.0 mm. Integument shining black except for segments 2–6 of antennal funicle reddish yellow and scape, funicular segments 1 and 7, club and tarsi dark brown. Rostrum in dorsal view with moderately distinct protuberance at antennal insertion. Antennae with globose scape, as long as wide and slightly shorter than first funicular segment, which is as robust as scape, 1.5× longer than wide and distinctly more robust than others, cylindrical, slightly shorter than second segment, which is 3.0× longer than wide, third and fourth segments respectively 2.2× and 1.6× longer than wide, fifth and sixth segments as long as wide, seventh segment transverse and poorly separated from club. Eyes convex. Prothorax transverse (Pw/Pl 1.50), subconical, widest, and with rounded sides in basal half; pronotum with rectilinear anterior margin, with sparse indistinct shallow small punctures, intervals between punctures large, with moderately shining surface and distinct microsculpture. Elytra distinctly globose, almost as long as wide (El/Ew 0.93), with humeri not pronounced, at base slightly wider than pronotum, widest just behind middle and distinctly wider than pronotum (Ew/Pw 1.55), distinctly convex; striae with small and nearly indistinct punctures widely separated from each other by wide intervals placed on the same plain as interstriae, the latter distinctly wider than striae, completely flat and without scales. Metafemora distinctly globose (l/w 2.5). Pro- and mesotibiae with small short uncus. First tarsomere 3.0× longer than wide, second tarsomere 1.5× longer than wide, onychium short, 2.3× longer than wide, claws long, single. Body of penis slightly wider at base, then with parallel sides to near apex, with distinctly blunted tip, with couple of long subtle sclerites bearing multiple, very small, more sclerotized spines and ending at orifice.

Variability. Length 0.8–1.1 mm. The width of the elytra varies a little also in the same population (El/Ew 0.92–1.01) as well as in the depth of the punctures of the elytral striae, which therefore appear more or less distinct.

*Etymology*. The name of this species highlights the rounded shape of the elytra.

*Comparative notes*. This taxon differs from the other species with a single claw by the rounded elytra, which are almost as long as wide, the punctures of the pronotum sparser and shallower, the elytral striae usually less demarcated, and the third tarsomere distinctly wider than second tarsomere. It is noteworthy that the type locality of this species is the same of that of *R. levipennis* and *R. pilosulus*.

*Biological notes*. This species was collected by E. Colonnelli on *Acacia* sp. and *Vachellia karroo* (Hayne) Banfi & Galasso (originally *Acacia karoo* Hayne) (Caesalpinioideae). It was also collected on *Vachellia* by J. Haran.

*Distribution*. South Africa (Mpumalanga, Limpopo, KwaZulu-Natal, Eastern Cape, Western Cape).

**D. *Rhamphus hispidulus* group** (one tarsal claw, integument with scales on dorsum).

13. ***Rhamphus hispidulus***  **sp. nov.** (Figure 4d)

LSID urn:lsid:zoobank.org:act:90A6A1F6-BB42-40CD-AAA3-93833492BD61

*Type locality*. Port St. Johns (Eastern Cape, South Africa).

*Type series*. Holotype ♀: “Port St. John[s]. Pondoland. May 15-31. 1923./S. Africa. R.E. Turner. Brit. Mus. 1923–332” (BMNH). Paratypes: “Port St. John[s]. Pondoland. 1-17 Mar. 1924./S. Africa. R.E. Turner. Brit. Mus. 1924–177” (1, RCPC); “SOUTH AFRICA: E Cape, -5 Km E Port St. Johns, 31.36.58S 29.34.61E, 8/9.XI.2006 - E. Colonnelli/Acacia sp. on leaves [green card]” (1, ECPC).

*Diagnostic description*. Holotype. Length 1.0 mm. Integument shining black except for funicular segments of antennae being reddish (scape and club blackish). Rostrum in dorsal view with moderately distinct protuberance at antennal insertion. Antennae with scape and first funicular segment globose and distinctly more robust than others, second segment 2.8× longer than wide, third and fourth segments 1.4× longer than wide, fifth–seventh segments transverse. Eyes convex. Prothorax subconical, transverse (Pw/Pl 1.67), widest just before middle, with rounded sides; pronotum with rectilinear anterior margin, with sparse, shallow, small punctures, intervals between punctures distinctly wider than punctures, and with moderately shining surface and distinct microsculpture. Elytra subelliptical, moderately longer than wide (El/Ew 1.25), with slightly pronounced humeri, at base slightly wider than pronotum, widest at middle (Ew/Pw 1.32), weakly convex; striae with distinct punctures being separated from each other by narrow intervals placed on a plain slightly lower than that of interstriae, the latter as wide as striae, slightly convex and covered with erect, subtle (l/w 15–20), whitish hair-like scales. Metafemora distinctly globose (l/w 2.5). Pro- and mesotibiae with small uncus (l/w 3). First tarsomere 3.5× longer than wide, second tarsomere 1.4× longer than wide, onychium short, 2.8× longer than wide, with single claw.

Variability. Length 0.9–1.0 mm. No significative differences between the three specimens of the type series.

*Etymology*. The name of this species refers to the erect hair-like scales on the elytral interstriae.

*Comparative notes*. This taxon is immediately separable from the other species with a single claw, in particular from the more closely related *R. indifferens*, by the erect setae on the elytra. However, also in no other species with two claws and with pubescent elytral vestiture are the setae erect.

*Biological notes*. This species was collected on *Acacia* sp. (det. E. Colonnelli), probably currently belonging to the genus *Vachellia*. *Distribution*: South Africa (Eastern Cape).

*Distribution*. South Africa (Eastern Cape).

#### 3.1.3. Key to the Species of *Rhamphus* from Southern Africa

1. Tarsi with two claws…………………………………….……………………………………2

- Tarsi with one claw……………………………………………………………..……………11

2. Elytra and pronotum without scales (Figure 1)……………………………………………3

- Elytra and pronotum with recumbent to suberect hair-like to rectangular scales (Figure 2 and Figure 3)…………………………………………………………………………………5

3. Pronotum with sparse punctures, with intervals between punctures wider than diameter of punctures…………………………………………..……….……………………..4

- Pronotum with dense punctures, with intervals between punctures narrower than diameter of punctures……………………………………………….*namibicus* Korotyaev

4. First tarsomere of metatarsi very long (l/w 6). Second tarsomere 1.5× longer than wide. Onychium 8× longer than wide. Punctures of pronotum deeper and broader; intervals between punctures 1.0–1.5× wider than punctures. Length 1.2–1.3 mm (Figure 1b)………………………………………………………...…………2. *longitarsis* sp. nov.

- First tarsomere of metatarsi shorter (l/w < 4). Second tarsomere as long as wide. Onychium 5× longer than wide. Punctures of pronotum shallow and very small; intervals between punctures 2–2.5× wider than punctures. Length 0.8–1.0 mm (Figure 1a)………………………………..…………………………………...….…1. *glaber* sp. nov.

5. Scales of dorsum rectangular to lanceolate, not hair-like, whitish (Figure 3d)…………………………………………………… 9. *squamidorsum* sp. nov.

- Scales of dorsum hair-like, whitish or blackish, sometimes almost transparent (Figure 2 and Figure 3a–c)……………………………………………………………………………..6

6. Dorsal vestiture with short, recumbent, hair-like scales well visible on pronotum but almost indistinct on elytra. Length 1.3–1.5 mm (Figure 2b)…4. *densepunctatus* sp. nov.

- Dorsal vestiture with longer, subrecumbent to suberect hair-like scales, well visible on both pronotum and elytra (Figure 2a,c and Figure 3a–c)……………….………………….7

7. Length 1.0–1.5 mm. Scape dark brown, darker than funicular segments. Dorsal integument shiny. Elytra on disc moderately convex (Figure 2a,c)…….…………………….8

- Length 1.5–2.2 mm. Scape as reddish as funicular segments. Dorsal integument opaque. Elytra on disc distinctly flat (Figure 3a,b).……………….……………………10

8. Elytra shorter, slightly longer than wide (El/Ew 1.12) (Figure 3c)…8. *obesulus* sp. nov.

- Elytra longer (El/Ew 1.21–1.32) (Figure 2a,c)……….……………………………..………9

9. Pronotum subrounded, moderately wider than long (Pw/Pl 1.40). Elytra with slightly rounded sides, somewhat longer than wide (El/Ew 1.32). Hair-like scales on pronotum brown, short, poorly visible (Figure 2a)…….…………………3. *pilosulus* sp. nov.

- Pronotum subconical, distinctly transverse (Pw/Pl 1.70). Elytra with moderately rounded sides, slightly longer than wide (El/Ew 1.21). Hair-like scales on pronotum white, long, well visible (Figure 2c)………………..……………………5. *scaber* sp. nov.

10. Body smaller (length 1.5–1.6 mm). Sides of elytra less rounded. Antennae shorter, funicle with second segment twice as long as wide and third segment 1.5× longer than wide (Figure 3a)……………………………………………….…6. *carinatus* sp. nov.

- Body larger (length 2.0–2.2 mm). Sides of elytra more rounded. Antennae longer, funicle with second segment 3.5× longer than wide, and third segment 2.5× longer than wide (Figure 3b)……………………………………………………………7. *gigas* sp. nov.

11. Elytra with erect seta-like scales. Pronotum with sparse punctures (Figure 4d)……………………………………………………13. *hispidulus* sp. nov.

- Elytra without seta-like scales. Pronotum with sparse to moderately dense punctures (Figure 4a–c)………………….……………………………………………………………….12

12. Elytra rounded to suboval, as long as or slightly shorter than wide (El/Ew 0.92–1.01). Pronotum with almost indistinct, very sparse, and very small punctures in basal two thirds. Third tarsomere distinctly wider than second tarsomere. Onychium 3× longer than wide (Figure 4c)…………………….……………12. *globipennis* sp. nov.

- Elytra elliptical, longer than wide (El/Ew 1.19–1.33). Pronotum with distinct, denser and slightly broader punctures. Third tarsomere moderately wider than second tarsomere. Onychium longer, 4× longer than wide (Figure 4a,b)………………..…………13

13. Length 0.7–0.9 mm. Pronotum subrounded, slightly wider than long (Pw/Pl 1.10–1.15). Punctures of elytral striae sparser, their diameter shorter than intervals between punctures (Figure 4a)….………….…………………………10. *levipennis* sp. nov.

- Length 1.0–1.2 mm. Pronotum subconical, distinctly transverse (Pw/Pl 1.45–1.50). Punctures of elytral striae denser, their diameter longer than intervals between punctures (Figure 4b)…………………………….………………………11. *indifferens* sp. nov.

## 4. Discussion

Prior to this paper, just one species, *Rhamphus namibicus*, based on a single specimen, had been described from Southern Africa [5]. However, Marshall [2] wrote that in his collection he possibly had new species but never described them. Therefore, it was not completely unexpected that we could find new species, even though a targeted collection of the genus was never carried out. It is worth noting that the genus *Rhamphus* has never really attracted weevil students so has always been poorly studied, mainly due to the species’ small size, which often is less than one millimeter in length.

The host plants of the southern African species seem to belong mainly to pan-tropical tribes of Caesalpinioideae and Detarioideae, which are two large, closely related subfamilies of Leguminosae, very recently subject to comprehensive revision [17,18]. It is noteworthy that in the Caealpinioideae [17], native species of the true genus *Acacia* are lacking in continental Africa. In this region the subfamily is represented by many indigenous species but also by several cultivated species (mainly originating from Australia); it should be assumed that many other species await discovery. The same is possibly true for the Australian continent, where the recognized species of *Acacia s.s.* already total more than one thousand [17].

Most of the species herein described may be clearly distinguished by good characters, e.g., the sculpture and the shape of pronotum and elytra, the presence or absence of vestiture, and the shape and the length of the antennal segments and the tarsal claws. On this basis and on the understanding that this is not a phylogenetic study, it was possible to separate four informal morphological groups: two groups with two more or less appendiculate tarsal claws, with glabrous or setose integument (which we here named, respectively, “*glaber*” and “*pilosulus*”), and two groups with a single claw, again with glabrous or setose integument (which we here named, respectively, “*levipennis*” and “*hispidulus*”). Usually, the species of the two latter groups have a less strongly punctured pronotum on the basal two thirds. We do not know *R. namibicus*: it was not possible to find and examine the holotype of this species in the collections of the Zoological Museum of Humboldt University, Berlin; however, by Korotyaev’s detailed original description [5], we can summarize that it lacks a pubescent vestiture and has two tarsal claws. By these two characters we can include this taxon into our *R. glaber* group, differing from *R. glaber* from Eastern South Africa by the sculpture of the pronotum, with denser and deeper punctures.

The relationships between the species from Southern Africa and those from the other regions is unknown. The African species seem to feed mainly on Caesalpinioideae, so it is possible that they are more related to the Australian species than to the central and northern Palaearctic ones, which live on clearly unrelated families of plants.

Many species of *Acacia s. s.* were introduced to countries around the world from the 19th century onwards as ornamentals, as hedges, to stabilize sand dunes, for timber or fuel, etc. Some of them have become pernicious invasive weeds [19]. Might it be possible that some Australian species were introduced to southern Africa with host plants, as happened with *R. kiesenwetteri* Tournier, 1873, casually described from specimens collected in Sicily but native to north Africa [15].

We asked Fiona Impson and Terence Olckers, two well-known experts in the biological control of *Acacia* s.l. introduced to South Africa from Australia, if they had collected *Rhamphus* specimens. Both of them told us that they never collected *Rhamphus* during their studies. Moreover, we tried to compare our species with the northern and central African and Australian species. We were able to study about 200 identified and unidentified specimens from Africa and Australia, and we did not find specimens belonging to species that we have here described. All of them have two tarsal claws and rarely have distinct hair-like scales, but these are recumbent and arranged in two to three rows. However, it is noteworthy that the single species described on a single specimen from the Oriental region (*R. medvedevi* Korotyaev, 1984, from Sri Lanka) has a single claw [5] and that one of the two species described from Japan (*R. hisamatsui* Chûjô & Morimoto, 1960) has a dorsal vestiture composed of suberect hair-like scales [16]. This species was placed in a new subgenus (*Trichorhamphus* Korotyaev, 1984) [16], which was subsequently considered as a synonym of *Rhamphus* [6] because it “cannot be maintained until a thorough revision of the world species of the first probably polyphiletic genus has been done”. We completely agree with Colonnelli’s sentence [6] and for the moment prefer to maintain a division of this genus into groups and not into subgenera. This applies especially to the species with a single claw, which is a character that defines some genera in Curculionidae, such as *Stereonychus* Suffrian, 1854 (Curculioninae, Cionini), and *Mononychus* Germar, 1823 (Ceutorhynchinae, Mononychini). In this regard, it will be crucial to establish whether *R. medvedevi* from Sri Lanka is phylogenetically related or not to the African species.

No southern African species seems similar to the very small species related to *R. kiesenwetteri* from Northern and Central Africa, which forms another distinct group treated by Voss [3] as his genus *Rhamphonyx* and by Korotyaev [16] as his subgenus *Nanorhamphus*, both taxa again rightly synonymized with *Rhamphus* by Colonnelli [6]. On the contrary, the species of the *R. glaber* group seem the closest to the Palaearctic species related to *R. oxyacanthae* and *R. pulicarius*, which however have the tarsal claws without appendices.

## Data Availability

All data used in this study are based on dried insect specimens deposited in publicly accessible institutional depositories (listed in Section 2.4) or in depositories of our colleagues (ibid.). All data used in this study are not subject to any legal or commercial restriction.
